# Analysis of HIV Drug Resistance Mutations Along With Treatment Outcomes of Efavirenz‐Based Regimens in Patients With V179D/E Mutations in Guangzhou, China

**DOI:** 10.1155/cjid/2598894

**Published:** 2026-08-18

**Authors:** Xingrong Zheng, Yunfei Xiao, Yeqiong Zhang, Peipei Wang, Ying Zhang, Lili Li, Yue Zheng, Xiaodi Li, Dabiao Chen, Xiaohua Yang, Zhanlian Huang

**Affiliations:** ^1^ Department of Infectious Diseases, Third Affiliated Hospital of Sun Yat-Sen University, Guangzhou, Guangdong, China, zssy.com.cn; ^2^ Guangdong Key Laboratory of Liver Disease Research, Third Affiliated Hospital of Sun Yat-Sen University, Guangzhou, Guangdong, China, zssy.com.cn

**Keywords:** antiretroviral therapy, drug resistance mutations, efficacy, HIV, V179D/E mutation

## Abstract

**Aim:**

To analyze the prevalence of human immunodeficiency virus type 1 (HIV‐1) drug resistance mutations (DRMs) and compare the therapeutic outcomes of different antiretroviral treatment regimens in newly diagnosed HIV‐1‐infected patients with the V179D/E mutation.

**Methods:**

This study included newly diagnosed HIV‐1‐infected individuals in Guangzhou from 2020 to 2024. Peripheral venous blood samples were collected, and viral RNA was extracted. The *pol* region of HIV genome was amplified and sequenced. Genotypic resistance testing was performed on the obtained *pol* gene sequences, and the prevalence of DRMs was analyzed. The efficacy of efavirenz (EFV)‐based and non‐EFV‐based regimens in patients with V179D/E mutation was compared using cumulative failure rate curves.

**Results:**

Of 740 enrolled patients who underwent baseline resistance testing, 198 (26.76%) harbored DRMs, of whom 59 (7.97%) exhibited pretreatment drug resistance (PDR). The predominant HIV subtypes were CRF07_BC and CRF01_AE. Non‐nucleoside reverse transcriptase inhibitors (NNRTIs) were associated with a DRM prevalence rate of 22.16% (*n* = 164/740), with common mutation sites being V179E (45.96%, *n* = 91/198) and V179D (12.12%, *n* = 24/198). 58.9% of patients with V179D/E mutations received EFV‐based regimens (EFV group, *n* = 66/112), while 41.1% (46/112) used integrase strand transfer inhibitors (INSTIs) or protease inhibitors (PIs)‐based regimens (non‐EFV group, *n* = 46). Besides, the EFV group exhibited significantly higher treatment failure rates than the non‐EFV group (*p* = 0.0131).

**Conclusion:**

The prevalence of DRMs in newly diagnosed HIV‐1‐infected individuals in Guangzhou associated with NNRTIs is 22.16%, primarily driven by the V179D/E mutation. EFV‐based treatments were associated with higher cumulative virological failure rates in patients with V179D/E mutation.

**Key Messages**
• What is already known on this topic?
◦ To our knowledge, there is a lack of statistical data on DRMs in HIV due to previous PDR studies excluding patients with potential resistance mutations, and cohort studies specifically evaluating EFV’s effectiveness in patients with the V179D/E mutation are also currently lacking.
• What this study adds?
◦ Our study shows that the prevalence of DRMs in newly diagnosed AIDS individuals in Guangzhou associated with NNRTIs is 22.16%, primarily driven by the V179D/E mutation. EFV‐based treatments were associated with higher cumulative virological failure rates in patients with V179D/E mutation.
• How this study might affect research, practice, or policy?
◦ The results indicate the need for close follow‐up and prompt regimen adjustments upon resistance detection.

## 1. Introduction

Antiretroviral therapy (ART) is now understood to be highly effective in controlling viral replication in human immunodeficiency virus (HIV)‐infected patients, reducing complications, mortality, and transforming acquired immune deficiency syndrome (AIDS) into a manageable chronic disease [1]. The past few years have witnessed substantial advancements in optimizing treatment protocols, enhancing drug resistance surveillance, and determining the optimal timing for initiating therapy [2, 3]. However, with increasing treatment duration and a growing population of treated individuals, the rising prevalence of HIV drug resistance undermines the effectiveness of ART in controlling HIV infection. The emergence of drug‐resistant HIV strains also poses the risk of treatment failure and the potential spread of new resistant variants, thereby increasing the burden on healthcare systems [4].

ART exerts selective pressure on HIV, leading to the emergence of mutations within its genetic sequence, which confer resistance to one or more antiretroviral drugs. These drug resistance mutations (DRMs) encompass a spectrum of resistance levels, comprising potential, low‐level, intermediate, and high‐level resistance mutations, ultimately leading to treatment failure [5, 6]. Pretreatment drug resistance (PDR) refers to the presence of mutations in the HIV viral strain that confer resistance to one or more antiretroviral drugs (ARVs) prior to the initiation of antiretroviral therapy (ART). However, PDR data from previous studies did not include potential resistance mutations [7, 8]. Currently, there is a lack of statistical data on DRMs in HIV. Furthermore, previous research mainly focused on men who have sex with men (MSM) populations, reporting that 9.3% of individuals exhibited resistance to non‐nucleoside reverse transcriptase inhibitors (NNRTIs), with the K103N/S mutation being the most prevalent at 7.4% [9]. There is currently a lack of detailed data on DRMs in heterosexual (HES) populations, as well as comparative analysis elucidating the similarities and differences in drug resistance between MSM and HES populations. Indeed, a comprehensive understanding of the DRM landscape, combined with enhanced drug resistance monitoring among high‐risk groups, can help optimize ARV treatment regimens and reduce the risk of drug resistance transmission.

Efavirenz (EFV) has been one of the most widely used NNRTIs in global HIV treatment programs. Despite its challenges regarding drug resistance and central nervous system side effects, EFV remains extensively used in resource‐limited regions and among populations who cannot access better alternatives due to its low cost and extensive historical usage experience. The V179D/E mutation primarily affects sensitivity to NNRTIs, particularly EFV and nevirapine (NVP) [10]. Moreover, the presence of the V179D/E mutation can reportedly increase the risk of subsequent resistance, especially with continued use of NNRTIs, potentially leading to the emergence of higher‐level resistance mutations [11]. Timely switching to integrase strand transfer inhibitors (INSTIs) or protease inhibitors (PIs) can mitigate the accumulation of resistance and improve long‐term viral suppression rates [12, 13]. Current international guidelines do not provide specific recommendations for the management of the V179D/E mutation. However, based on established resistance mechanisms and clinical experience, it is generally advised to avoid using NNRTIs and prioritize high‐barrier drugs [14, 15]. Nonetheless, in economically underdeveloped areas, EFV remains widely used as a first‐line treatment regimen. Cohort studies specifically evaluating the effectiveness of EFV in patients with the V179D/E mutation are currently lacking, emphasizing the need for a real‐world study to elucidate the efficacy of different treatment regimens for the V179D/E mutation.

This study analyzed HIV‐1 subtypes, DRMs, and the therapeutic outcomes of different treatment regimens in ART‐naïve individuals newly diagnosed with HIV‐1 infection harboring the V179D/E mutation. Importantly, our results provide the theoretical basis for the development of localized ART treatment strategies.

## 2. Methods

### 2.1. Patient Screening and Data Collection

This retrospective cohort study was conducted at the Third Affiliated Hospital of Sun Yat‐sen University, including newly reported HIV‐1‐infected patients from May 2020 to September 2024. The inclusion criteria were as follows: (1) patients newly diagnosed with HIV‐1 infection with laboratory confirmation; (2) ART‐naïve status; and (3) enrolled patient provided general informed consent for the utilization of their clinical information in scientific research. Exclusion criteria were as follows: (1) history of ART use; (2) absence of PDR testing or missing clinical data; (3) nonadherence to follow‐up protocols after treatment initiation. Patients included were followed up every 3 months, during which biochemical tests were conducted. Besides, HIV RNA levels and CD4 cell counts were assessed every 6 months. Demographic and epidemiological information was obtained from the hospital information system (HIS). MSM (men who have sex with men) was defined as self‐identified males reporting sexual contact with men within the past 12 months, while HES referred to patients reporting HES contact as the sole risk factor with no concurrent risk behaviors. All patients were followed up for a minimum of 24 weeks (6 months) after ART initiation, with the primary endpoint assessed at this time point and data collection ending on March 1, 2025. Those continuing treatment beyond 48 weeks were subsequently followed up to 96 weeks, with scheduled visits at weeks 4, 12, 24, and 48, and every 24 weeks thereafter for patients with extended follow‐up. This study was conducted in accordance with the Declaration of Helsinki and was approved by the Ethics Committee of the Third Affiliated Hospital of Sun Yat‐sen University with patient information anonymized for analysis (II2025‐028‐01).

### 2.2. Nucleic Acid Extraction, Amplification, and Sequencing

EDTA‐anticoagulated whole blood samples (6–8 mL) were collected immediately after initial confirmation of diagnosis, and plasma was separated and stored at −80°C for subsequent use. RNA extraction was performed using the QIAamp Viral RNA Mini Kit (Qiagen, Germany) from 140 μL of plasma samples. The HIV genome contains three structural genes (gag, pol, and env). For drug resistance testing, we amplified the full‐length protease region (1–99 AA), the first 300 amino acids of the reverse transcriptase region, and the full‐length integrase region (1–288 AA), all of which are located within the pol gene (1316 bp, corresponding to positions 2147–3462 of the reference strain HXB2), using reverse transcription PCR and nested PCR. The PCR reagents were from Takara Bio Inc. (Japan). The PCR products were verified by 1% agarose gel electrophoresis, subsequently purified and sequenced by Hangzhou QK Biotechnology Co., Ltd. The amplification sites, primer names, and sequence information are presented in the supporting Table S1.

### 2.3. Subtype Analysis

Sequencing data were assembled and corrected using Sequencher 5.4.6 software. Sample sequences were aligned with international reference strains (from the Los Alamos National Laboratory HIV Sequence Database) using BioEdit 7.2.0 software. A Neighbor‐Joining phylogenetic tree was constructed using MEGA 7.0 software (Kimura 2‐parameter model, with 1000 bootstrap replicates). Subtypes were determined based on clustering with reference strains, using a bootstrap value threshold of ≥ 75%. Sequences that did not cluster with reference strains were considered unique recombinant forms (URFs). Recombination types were analyzed using the Jumping Profile HMM (jpHMM) online tool (https://jphmm.gobics.de).

### 2.4. Drug Resistance Analysis

Sequences were submitted to the Stanford University HIV Drug Resistance Database (https://hivdb.stanford.edu/) for analysis of DRMs and resistance levels for nucleoside reverse transcriptase inhibitors (NRTIs), NNRTIs, and PIs, covering a total of 20 ARV drugs. Raw sequence data were not available for public deposition as testing was performed by laboratory of Guangdong Provincial Quality Control Center for Diagnosis and Treatment of AIDS and Hepatitis C; only interpreted resistance profiles were provided to the study team. The corresponding Stanford HIV Database (HIVDB) version numbers ranged from HIVDB 8.9‐1 (released on October 15, 2019) to HIVDB 9.6 (released on March 9, 2024). Resistance testing was performed for all samples with baseline viral load > 200 copies/mL, and genotyping was conducted based on the obtained sequences. In this study, all enrolled patients had baseline viral loads > 200 copies/mL, and no samples failed resistance testing. Resistance was defined as the presence of any DRM, including high‐, intermediate‐, or low‐level or potential resistance mutations. According to the Stanford HIV‐1 Drug Resistance Database scoring system, high‐level resistance was defined as a score of ≥ 60 points, intermediate resistance as 30–59 points, low‐level resistance as 15–29 points, and potential resistance as 10–14 points [16]. The 20 drugs analyzed included atazanavir (ATV), darunavir (DRV), fosamprenavir (FPV), lopinavir (LPV), indinavir (IDV), nelfinavir (NFV), saquinavir (SQV), tipranavir (TPV), lamivudine (3TC), abacavir (ABC), zidovudine (AZT), stavudine (D4T), didanosine (DDI), emtricitabine (FTC), tenofovir (TDF), efavirenz (EFV), etravirine (ETR), nevirapine (NVP), rilpivirine (RPV), and doravirine (DOR).

### 2.5. Statistical Analysis

Data were collected using EpiData 3.0, organized with Excel 2016, and analyzed using SPSS 20.0 and Graphpad Prism software. Categorical variables were reported as frequencies (proportions) and analyzed using chi‐square tests or Fisher’s exact tests, as appropriate. Continuous variables were described using mean ± standard deviation (SD) or median (interquartile range, IQR) and comparisons between groups were conducted using *t*‐tests or Wilcoxon rank‐sum tests. Statistical significance was defined as a two‐sided *p* value less than 0.05. The differences between EFV and non‐EFV treatment in patients harboring the V179D/E mutation were compared using cumulative failure rate curves.

## 3. Results

### 3.1. Clinical Characteristics

**FIGURE 1:**
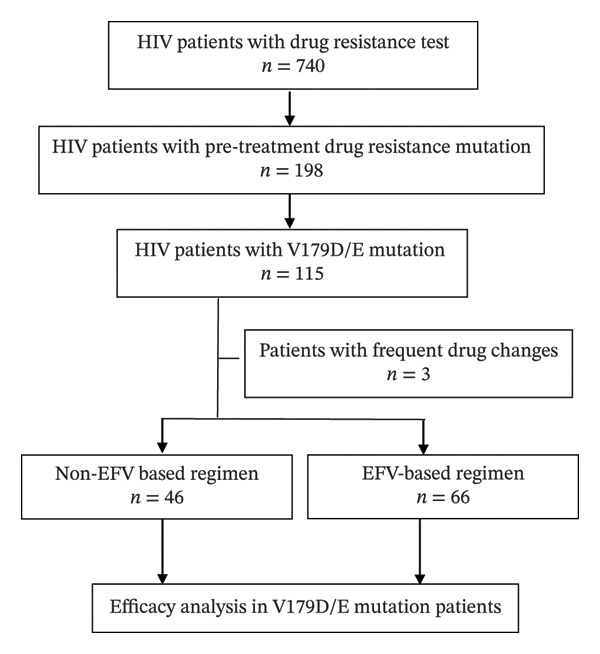
Flowchart of the study.

A total of 740 newly reported HIV‐1‐infected patients with baseline drug resistance testing and HIV subtype analysis (prior to ART initiation) were identified in the outpatient clinic, comprising MSM (*n* = 536) and HES (*n* = 204) (Figure 1). The predominant HIV subtypes in both groups were CRF07_BC (48.32% in MSM, 47.55% in HES), CRF01_AE (29.29% in MSM, 30.39% in HES), and CRF55_01B (10.26% in MSM, 8.33% in HES). Compared with the HES group, the MSM group was characterized by younger age, non‐marital status, and higher educational attainment (college degree or above). Moreover, the MSM group exhibited higher baseline absolute CD4 and CD8 counts than the HES group (Table 1).

**TABLE 1:** Baseline characteristics of enrolled patients between MSM and HES groups.

Characteristics	Total (*n* = 740)	MSM (*n* = 536)	HES (*n* = 204)	*p* value
Ages, median (IQR)	32 (26, 43)	29 (25, 37)	43 (34, 55)	< 0.001
Gender, *n* (%)				< 0.001
Male	689 (93.11%)	536 (100%)	153 (75.00%)	
Female	51 (6.89%)	0 (0.00%)	51 (25.00%)	
Marriage, *n* (%)				< 0.001
Married	180 (24.32%)	86 (16.04%)	94 (46.08%)	
Others	560 (75.68%)	450 (83.96%)	110 (53.92%)	
Education, *n* (%)				< 0.001
College	366 (49.46%)	314 (58.58%)	52 (25.49%)	
Below college	374 (50.54%)	222 (41.42%)	152 (74.51%)	
CD4+, median (IQR)	297.50 (188.50, 414.00)	316.50 (210.75, 436.00)	249.00 (144.75, 338.00)	< 0.001
CD8+, median (IQR)	1072.00 (729.50, 1462.00)	1109.50 (794.75, 1516.50)	948.00 (618.25, 1204.00)	< 0.001
HIV Subtype, *n* (%)				0.002
CRF07_BC	356 (48.11%)	259 (48.32%)	97 (47.55%)	
CRF01_AE	219 (29.59%)	157 (29.29%)	62 (30.39%)	
CRF55_01B	72 (9.73%)	55 (10.26%)	17 (8.33%)	
CRF59_01B	21 (2.84%)	16 (2.99%)	5 (2.45%)	
B	34 (4.59%)	28 (5.22%)	6 (2.94%)	
CRF08_BC	16 (2.16%)	4 (0.75%)	12 (5.88%)	
other	22 (2.97%)	17 (3.17%)	5 (2.45%)	
DRMs (Total), *n* (%)			0.049	
Yes	198 (26.76%)	154 (28.73%)	44 (21.57%)	
No	542 (73.24%)	382 (71.27%)	160 (78.43%)	
DRMs (NRTIs), *n* (%)				0.375
Yes	21 (2.84)	17 (3.17%)	4 (1.96%)	
No	719 (97.16%)	519 (96.83%)	200 (98.04%)	
DRMs (NNRTIs), *n* (%)				0.104
Yes	164 (22.16%)	127 (23.69%)	37 (18.14%)	
No	576 (77.84%)	409 (76.31%)	167 (81.86%)	
DRMs (PIs), *n* (%)				0.606
Yes	16 (2.16%)	13 (2.43%)	3 (1.47%)	
No	724 (97.84%)	523 (97.57%)	201 (98.53%)	
DRMs (INSTIs), *n* (%)				0.303
Yes	8 (1.08%)	4 (0.75%)	4 (1.96%)	
No	732 (98.92%)	532 (99.25%)	200 (98.04%)	

### 3.2. Prevalence of DRMs

According to the analysis from the Stanford University HIV Drug Resistance Database, among the 740 HIV‐infected individuals, 198 presented with baseline DRMs, yielding an overall prevalence rate of 26.76% (*n* = 198/740). Specifically, the prevalence of DRMs was 2.16% (*n* = 16/740) for PIs, 2.84% (*n* = 21/740) for NRTIs, 22.16% (*n* = 164/740) for NNRTIs, and 0.54% (*n* = 4/740) for INSTIs (Table 1). Moreover, 1.22% (9/740) of patients exhibited resistance to two or more classes of drugs. The prevalence of PDR, excluding potential resistance mutations, was 7.97% (*n* = 59/740). The overall DRM prevalence was slightly higher in the MSM population than in the HES group (Table 1). However, no significant differences in DRM prevalence were observed between the two groups after stratification by drug class (Figure S1A). Moreover, the drug resistance analysis revealed no significant differences in resistance rates across different genetic subtypes between the MSM and HES groups (Figure S1B).

### 3.3. Distribution of Mutation Sites and Genotypes of DRMs

**FIGURE 2:**
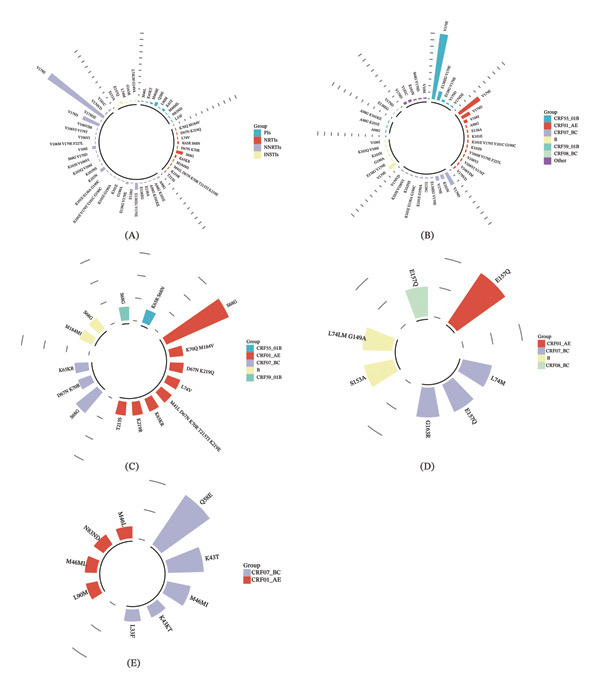
Grouped radial bar chart of mutation sites in different drugs and subtypes. (A) Grouped radial bar chart of mutation sites in different drugs; (B) grouped radial bar chart of mutation sites in NNRTIs among subtypes; (C) grouped radial bar chart of mutation sites in NRTIs among subtypes; (D) grouped radial bar chart of mutation sites in INSTIs among subtypes; (E) grouped radial bar chart of mutation sites in PIs among subtypes.

Among the cases with DRMs (*n* = 198), the common mutation sites for NNRTIs were V179E (45.96%, *n* = 91/198) and V179D (12.12%, *n* = 24/198). The predominant mutation was S68G (4.55%, *n* = 9/198) for NRTIs, Q58E (2.53%, *n* = 5/198) for PIs, and E157Q (2.02%, *n* = 4/198) for INSTIs (Figure 2A). The DRM prevalence was 100% (*n* = 72/72) among individuals with the CRF55_01B subtype, all of whom exhibited the V179D/E mutation (Figure S1B), with two patients harboring the K65R and S68N mutations (Figure 2B,C). In contrast, the DRM prevalence for the CRF07_BC subtype was 12.64% (*n* = 45/356) (Figure S1B), with the most frequent mutation being V179D/E (NNRTI‐associated, *n* = 17/356, 4.78%), followed by K103N (NNRTI‐associated, *n* = 5/356, 1.40%) (Figure 2B), S68G (NRTI‐associated, *n* = 2/356, 0.56%) (Figure 2C), L74M (INSTI‐associated, *n* = 1/356, 0.28%) (Figure 2D), and Q58E (PI‐associated, *n* = 5/356, 1.40%) (Figure 2E). For the CRF01_AE subtype, the DRM prevalence was 24.66% (*n* = 54/219) (Figure S1B), with the V179D/E mutation being the most frequent (NNRTI‐associated, *n* = 31/219, 14.16%) (Figure 2B), alongside other mutations such as V106I (NNRTI‐associated, *n* = 3/219, 1.37%) (Figure 2B), S68G (NRTI‐associated, *n* = 5/219, 2.28%) (Figure 2C), E157Q (INSTI‐associated, *n* = 2/219, 0.91%) (Figure 2D), and L90M (PI‐associated, *n* = 1/219, 0.46%) (Figure 2E).

### 3.4. Drug Resistance Profiles Across Antiretroviral Agents

As shown in (Figure S2), in both MSM and HES populations, NNRTIs mainly exhibited potential resistance (17.35% vs. 14.71%) and high‐level resistance (2.99% vs. 1.47%), while NRTIs predominantly showed high‐level resistance (1.12% vs. 0.98%). In contrast, PIs primarily demonstrated low‐level resistance (1.12%) in MSM, INSTIs showed potential resistance (0.98%) in HES, and INSTIs mainly showed potential resistance (0.56% vs. 1.47%). Among these drug resistance categories, multiclass drug resistance was documented in both MSM and HES populations (Figure S2A). Besides, these findings indicated that DRMs were primarily driven by NNRTIs mutations, with potential resistance mutations being the most common (*n* = 123/164, 75%), followed by high‐level resistance mutations (*n* = 19/164, 11.59%). NRTI‐associated DRMs were mainly high‐level resistance mutations (*n* = 7/12, 58.33%), PI‐associated DRMs were predominantly low‐level resistance mutations (*n* = 8/16, 50%), and INSTI‐associated DRMs were mostly potential resistance mutations (*n* = 6/7, 85.71%) (Figure S2A). The distribution of common DRMs for each category is illustrated in (Figure S2B). Moreover, resistance‐associated mutations varied in prevalence and severity in both MSM and HES populations, with a higher number of mutation sites observed in the MSM population (supporting Table S2). These results highlight the need for clinicians to closely monitor drug efficacy, resistance, and adjust the treatment regimen during the use of NNRTIs and NRTIs by HIV patients.

### 3.5. Impact of V179D/E Mutation on NNRTI Treatment Efficacy and Potential Resistance

Within the study population, 115 patients were identified at baseline with the V179D/E mutation and 3 patients were excluded due to frequent drugs changes leaving 112 cases for statistical analysis (Figure 1). These individuals, exhibiting potential resistance, were initially treated with NNRTI, PI or INSTI‐based regimens. To further explore the therapeutic efficacy of different treatment regimens in patients with V179D/E mutations, the patient cohort was divided into two groups. Patients in the non‐EFV group received one of the following regimens: lopinavir/ritonavir (LPV/r) 500 mg twice daily plus tenofovir disoproxil fumarate (TDF) 300 mg plus lamivudine (3TC) 300 mg once daily, bictegravir/emtricitabine/tenofovir alafenamide (BIC/FTC/TAF) one tablet once daily, or dolutegravir/lamivudine (DTG/3TC) one tablet once daily (non‐EFV group, *n* = 46). Patients in the EFV group received a once‐daily regimen of tenofovir disoproxil fumarate (TDF) 300 mg plus lamivudine (3TC) 300 mg plus efavirenz (EFV) 400 mg or 600 mg (EFV group, *n* = 66). There were no significant differences between the two groups in terms of gender, age, treatment duration, baseline HIV RNA viral load, and baseline CD4 and CD8 levels (supporting Table S3).

**FIGURE 3:**
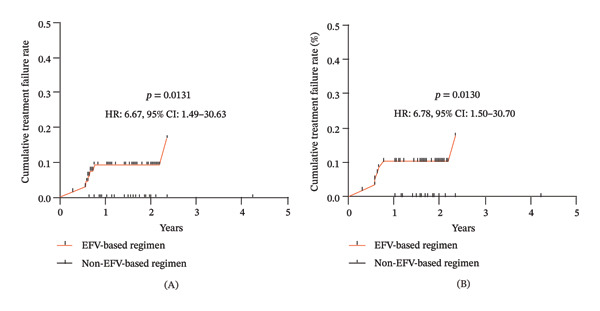
The cumulative drug treatment failure rate between patients using EFV‐ and non‐EFV‐based regimens. (A) Comparison of cumulative treatment failure rates between non‐EFV (*n* = 46) and EFV‐based (*n* = 66) regimen groups. (B) Comparison of cumulative treatment failure rates between non‐EFV (*n* = 41) and EFV‐based (*n* = 58) groups, excluding patients with <12 months of follow‐up and no virological failure.

Meanwhile, we further analyzed the virologic failure rates between the two groups. Virologic failure is defined as the persistent plasma viral load > 200 copies/mL after 24 weeks of ongoing antiretroviral therapy (ART) initiation or modification, or virologic rebound meaning the re‐emergence of viral load ≥ 200 copies/mL after achieving complete virologic suppression in patients who remain on ART. For patients with a single detectable viral load (50–200 copies/mL), a repeat test was performed within 4 weeks. If the repeat value remained ≥ 200 copies/mL, this was classified as confirmed virologic failure. In the EFV group, the median follow‐up duration was 1.86 years, with a minimum follow‐up duration of more than 6 months. Seven patients failed to achieve viral suppression at 4 months (*n* = 1), 6 months (*n* = 5), and 2 years (*n* = 1) of treatment (supporting Table S4). In the non‐EFV group, the median follow‐up time was 2.49 years. At 6 months of treatment, all patients exhibited undetectable HIV RNA levels until the last follow‐up. Comparing the cumulative treatment failure rates between the two groups revealed that the EFV group had a significantly higher cumulative failure rate than the non‐EFV group (*p* = 0.013, HR: 6.67, 95% CI: 1.49–30.63) (Figure 3A). After excluding patients with follow‐up duration of less than 12 months without virological failure, we compared the two groups again, resulting in 58 patients in the EFV group and 41 in the non‐EFV group. The results showed that patients in the EFV group still had a higher virological failure rate (*p* = 0.0130, HR: 6.78, 95%CI: 1.50–30.70) (Figure 3B). These results indicated that patients harboring potential resistance by the V179D/E mutation showed relatively poorer virological control when treated with an EFV‐based regimen. Clinically, close patient monitoring is recommended, and prompt adjustments to treatment regimens are indicated in the event of detected resistance.

## 4. Discussion

Herein, we analyzed the HIV‐1 subtypes, prevalence and characteristics of DRMs and the treatment outcomes of patients with the V179D/E mutation. The predominant genetic subtypes among newly diagnosed HIV patients in Guangzhou were CRF07_BC (48.11%), CRF01_AE (29.59%) and CRF55_01B (9.73%). Both MSM and HES groups were predominantly infected with CRF07_BC and CRF01_AE subtypes, while CRF08_BC was more prevalent in the HES population (5.88%), consistent with the literature [17, 18]. The prevalence of DRMs and PDR was 26.22% and 7.98%, respectively, indicating moderate resistance, which is consistent with studies conducted in other regions of China [19, 20].

Prior reports have focused on pretreatment drug resistance, excluding patients with potential resistance. However, growing evidence suggests that the prevalence of potential resistance is relatively high, highlighting the need for further research on differences in potential resistance across various populations [21, 22]. Therefore, this study analyzed individuals with potential resistance and revealed minor variations in the overall prevalence of DRMs between MSM and HES populations. We found higher prevalence of DRM in MSM, which may be related to suboptimal pre‐exposure prophylaxis or poor adherence to ART, a greater number of sexual partners and more complex sexual networks [23]. Similar to previous research, DRMs were mainly identified in CRF07_BC and CRF01_AE subtypes, whereas V179D/E was predominantly observed in CRF55_01B (an intrinsic feature) and CRF01_AE. Besides, the NRTI‐resistant population included a certain proportion of CRF55_01B and B subtypes, a novel finding potentially linked to subtype‐specific transmission dynamics, host behavioral factors, and ARV exposure [24, 25].

In our study, NNRTI‐associated resistance mutations were the most prevalent type of DRM (22.16%). Currently, NNRTI resistance has become a key issue in HIV antiviral therapy, and there are increasing reports indicating an upward trend in its resistance rate [26, 27]. In our cohort, V179D/E was the most prevalent NNRTI resistance mutation (58.08% of DRMs), conferring potential NNRTI resistance, consistent with previous reports in treatment‐naïve patients [26]. High‐level NNRTI resistance, the second most prevalent resistance phenotype, was detected in 2.57% (*n* = 19/740) of cases, underscoring its clinical significance. The mutation sites associated with high‐level resistance included K103N, V106M, and Y181C, with prevalence rates of 1.08%, 0.27%, and 0.27%, respectively, which are comparable to those reported in previous studies [22]. Within NRTIs, S68G emerged as the most frequent mutation. Although S68G does not directly confer resistance, it may act as a compensatory mutation for K65R, mitigating its replication defect and thereby promoting viral persistence under drug pressure [28]. This interaction may influence the selection of subsequent treatment regimens. Apart from the S68G mutation, other NRTI‐associated mutations were associated with high‐level resistance, including K65R and M184I/V. Resistance mutations to PIs and INSTIs were relatively rare in our results, with PIs primarily exhibiting low‐level resistance and INSTIs primarily showing potential resistance. However, with the widespread use of these drug classes, proactive resistance surveillance remains essential.

EFV is the most widely used NNRTI, and the V179D/E mutation is one of the most common mutations associated with NNRTI use. Therefore, studying the prevalence of the V179D/E mutation in ART‐naive HIV‐infected patients and comparing the efficacy of EFV‐based regimens can help elucidate the clinical significance of the HIV‐1 V179D/E mutation. In this study, the V179D/E mutation was primarily found in the CRF01_AE and CRF55_01B HIV recombinant forms, consistent with the literature [29, 30]. Although the level of resistance caused by the V179D/E mutation was low, this study found that EFV‐based treatment regimens yielded a higher cumulative failure rate. While previous research has indicated that EFV‐based treatment regimens may be less effective in patients with high viral loads (≥ 1,00,000 copies/mL) [10], our study did not further perform comparison of the impact of different viral load levels on these patients given that all patients with the V179D/E mutation had viral loads more than 10,000 copies/mL. Accordingly, prospective cohort studies are warranted to further confirm the research conclusions and provide more effective treatment regimens for such patients.

Our study has certain limitations that should be acknowledged. First, although this was a retrospective, single‐center study with a relatively small sample size, which limits the generalizability of the findings to the broader landscape of HIV within the country, it should be noted that our study population was predominantly young men who were MSM, making our findings particularly relevant to regions with similar demographic characteristic. Future prospective multicenter studies are essential to further validate our results. Second, the follow‐up period was relatively short for some patients, which may affect the assessment of the efficacy of different treatment regimens for patients with the V179D/E mutation. The efficacy of different regimens could not be assessed through cumulative failure rates. Future studies should include longitudinal monitoring of virological responses. Finally, our study did not investigate the basic mechanisms of DRMs and PDR, nor did it examine immunological aspects, emphasizing the need for more data to conduct a comprehensive analysis.

## 5. Conclusions

The prevalence of DRMs associated with NNRTIs was 22.16% in Guangzhou, primarily driven by the V179D/E mutation. Patients harboring the V179D/E mutation are associated with higher cumulative failure rates with EFV‐based treatments, necessitating close follow‐up and prompt regimen adjustments upon resistance detection.

## Author Contributions

Xingrong Zheng: data collection and analysis, wrote the original draft, and reviewed the manuscript. Yunfei Xiao: collected and organized clinical data, supervised the project, provided resources, and wrote the original draft. Yeqiong Zhang: data collection and formal analysis, provided resources, and reviewed and edited the manuscript. Peipei Wang: collected and organized data, conducted research, and reviewed the manuscript. Ying Zhang, Lili Li, Yue Zheng, and Xiaodi Li: organized data and follow‐up of patients, and reviewed and edited the manuscript. Dabiao Chen and Xiaohua Yang: designed and supervised the project, follow‐up of patients, provided resources, wrote the original draft, and reviewed and edited the manuscript. Zhanlian Huang: designed and supervised the research, obtained funding, follow‐up of patients, data validation, and wrote and reviewed the manuscript. Xingrong Zheng, Yunfei Xiao, and Yeqiong Zhang contributed equally to this work.

## Funding

This study was supported by the Science and Technology Program of Guangdong Province (KTPYJ2023002) and Prevention and Control of Emerging and Major Infectious Diseases‐National Science and Technology Major Project (project 2025ZD01904600, subproject 2025ZD01904602 and project 2025ZD01904900, subproject 2025ZD01904902).

## Ethics Statement

The study protocol was approved by Medical Ethics Committee of the Third Affiliated Hospital of Sun Yat‐sen University (II2025‐028‐01). Enrolled patient provided general informed consent for the utilization of their clinical information in scientific research.

## Consent

Please see the Ethics Statement.

## Conflicts of Interest

The authors declare no conflicts of interest.

## Supporting Information

Additional supporting information can be found online in the Supporting Information section.

## Supporting information


**Supporting Information 1** Figure S1. Distribution of DRMs among various classes of antiviral drugs and subtypes between MSM and HES populations in HIV. (A) DRMs in different classes of drugs; (B) DRMs in different HIV‐1 subtypes.

**Supporting Information 2** Figure S2. Distribution of the degree of drug resistance among different antiviral drugs. (A) Distribution of different degrees of drug resistance among various classes of antiviral drugs between MSM and HES populations; (B) various degrees of drug resistance among antiviral drugs.

**Supporting Information 3** Table S1. Details of the amplification sites, primer names, and sequence information.

**Supporting Information 4** Table S2. Distribution of genetic mutations and drug resistance in 198 pre‐treatment HIV patients in MSM and HES patients.

**Supporting Information 5** Table S3. Comparison of baseline characteristics of individuals with V179D/E mutation across different treatment regimens.

**Supporting Information 6** Table S4. Characteristics of patients with V179D/E mutation and drug resistances.

## Data Availability

Data are available on reasonable request.
