# Supplementary material for: Analysis of HIV Drug Resistance Mutations Along With Treatment Outcomes of Efavirenz‐Based Regimens in Patients With V179D/E Mutations in Guangzhou, China
Source: Can J Infect Dis Med Microbiol. 2026 Aug 18;2026:2598894. doi: 10.1155/cjid/2598894 (PMC13485334; doi:10.1155/cjid/2598894)
Supplement: Supplementary file 1 [file CJID-2026-2598894-s003.docx]

**Table S1. Details of the amplification sites, primer names, and sequence information**

| **Amplification sites** | **primer names** | **sequence** | **Location to HXB2** |
| --- | --- | --- | --- |
| Protease and reverse transcriptase regions | MAW26 | 5’- TTGGAAATGTGGAAAGGAAGGAC -3’ | 2028-2050 |
|  | RT21 | 5’- CTGTATTTCTGCTATTAAGTCTTTTGATGGG -3’ | 3539-3509 |
|  | PRO1 | 5’- CAGAGCCAACAGCCCCACCA -3’ | 2147-2166 |
|  | RT20 | 5’- CTGCCAGTTCTAGCTCTGCTTC -3’ | 3462-3441 |
|  | PROS3 | 5’- GCCAACAGCCCCACCA -3’ | 2151-2166 |
|  | RTAS | 5’- CTCAGATTGGTTGCAC -3’ | 2524-2539 |
|  | RTB | 5’- CCTAGTATAAACAATGAGACAC -3’ | 2946-2967 |
|  | PROC1S | 5’- GCTGGGTGTGGTATTCC -3’ | 2842-2826 |
|  | RT20S3 | 5’- GTTCTAGCTCTGCTTC -3’ | 3456-3441 |
| Integrase region | F1-IN | 5’- TTGGAGGAAATGAACAAGTAGATAAATTAGT -3’ | 4174-4204 |
|  | R1-IN | 5’- AATCCTCATCCTGTCTACTTGCCACACAATC -3’ | 5094-5064 |
|  | F2-IN | 5’- TTTTAGATGGAATAGATAAGGCYCAAGA -3’ | 4231-4258 |
|  | R2-IN | 5’- GCCACACAATCATCACCTGCCATCTGTTTT -3’ | 5074-5045 |
