# Supplementary material for: Analysis of HIV Drug Resistance Mutations Along With Treatment Outcomes of Efavirenz‐Based Regimens in Patients With V179D/E Mutations in Guangzhou, China
Source: Can J Infect Dis Med Microbiol. 2026 Aug 18;2026:2598894. doi: 10.1155/cjid/2598894 (PMC13485334; doi:10.1155/cjid/2598894)
Supplement: Supplementary file 2 [file CJID-2026-2598894-s004.docx]

**Table S2. Distribution of genetic mutations and drug resistance in 198 pre-treatment HIV patients in MSM and HES patients**

|  |  | **MSM** | | | **HES** | | |
| --- | --- | --- | --- | --- | --- | --- | --- |
| **Mutation Site** | **Total**  **(n)** | **Mutation (n)** | **Highest level of resistance** | **Resistant drugs** | **Mutation (n)** | **Highest level of resistance** | **Resistant drugs** |
| **NRTIs** | 21 | 17 |  |  | 4 |  |  |
| D67N, K70R | 1 | 1 | I | ABC, AZT, D4T, DDI, TDF | 0 | - | - |
| D67N, K219Q | 1 | 1 | L | ABC, AZT, D4T, DDI, TDF | 0 | - | - |
| K219R | 1 | 1 | P | AZT, D4T | 0 | - | - |
| K65KR | 2 | 2 | H | ABC, D4T, DDI, FTC, 3TC, TDF | 0 | - | - |
| K65R, S68N | 2 | 1 | H | ABC, D4T, DDI, FTC, 3TC, TDF | 1 | H | ABC, D4T, DDI, FTC, 3TC, TDF |
| K70Q, M184V | 1 | 1 | H | ABC, D4T, DDI, FTC, 3TC, TDF | 0 | - | - |
| L74V | 1 | 0 | - | - | 1 | H | ABC, DDI |
| M184MI | 1 | 1 | H | ABC, DDI, FTC, 3TC | 0 | - | - |
| M41L, D67N, K70R, T215TI, K219E | 1 | 1 | H | ABC, AZT, D4T, DDI, FTC, 3TC, TDF | 0 | - | - |
| S68G | 9 | 7 | S | - | 2 | S | - |
| T215S | 1 | 1 | L | AZT, D4T,DDI | 0 | - | - |
| **NNRTIs** | 164 | 127 |  |  | 37 |  |  |
| A98G | 2 | 2 | I | DOR, EFV, ETR, NVP, RPV | 0 | - | - |
| A98G, K101E | 1 | 1 | H | DOR, EFV, ETR, NVP, RPV | 0 | - | - |
| A98G, K101KE | 1 | 1 | H | DOR, EFV, ETR, NVP, RPV | 0 | - | - |
| E138A | 1 | 1 | L | ETR, RPV | 0 | - | - |
| E138EG | 1 | 0 | - | - | 1 | L | EFV, ETR, NVP, RPV |
| E138EG, V179E | 9 | 7 | L | EFV, ETR, NVP, RPV | 2 | L | EFV, ETR, NVP, RPV |
| E138G | 1 | 1 | L | EFV, ETR, NVP, RPV | 0 | - | - |
| E138G, V179E | 3 | 2 | L | EFV, ETR, NVP, RPV | 1 | L | EFV, ETR, NVP, RPV |
| G190A | 1 | 0 | - | - | 1 | H | EFV, ETR, NVP, RPV |
| K101E | 1 | 1 | I | DOR, EFV, ETR, NVP, RPV | 0 | - | - |
| K101E, G190A | 1 | 1 | H | DOR, EFV, ETR, NVP, RPV | 0 | - | - |
| K101E, V179T, Y181C, G190C | 1 | 0 | - | - | 1 | H | DOR, EFV, ETR, NVP, RPV |
| K101E, E138A, G190C | 1 | 1 | H | DOR, EFV, ETR, NVP, RPV | 0 | - | - |
| K103N | 7 | 7 | H | EFV, NVP | 0 | - | - |
| K103NS | 1 | 0 | - | - | 1 | H | EFV, NVP |
| K103Q, V106I | 1 | 1 | P | DOR, ETR, NVP, RPV | 0 | - | - |
| K103S, V106VI | 1 | 1 | H | EFV, NVP, | 0 | - | - |
| V106I | 5 | 4 | P | DOR, ETR, NVP, RPV | 1 | P | DOR, ETR, NVP, RPV |
| V106M, V179E, F227L | 1 | 1 | H | DOR, EFV, ETR, NVP, RPV | 0 | - | - |
| V106VI | 1 | 0 | - | - | 1 | P | DOR, ETR, NVP, RPV |
| V106VI, V179T | 1 | 0 | - | - | 1 | P | DOR, ETR, NVP, RPV |
| V106VIM | 1 | 1 | H | DOR, EFV, ETR, NVP, RPV | 0 | - | - |
| V179D | 21 | 11 | P | EFV, ETR, NVP, RPV | 10 | P | EFV, ETR, NVP, RPV |
| V179DE | 1 | 1 | P | EFV, ETR, NVP, RPV | 0 | - | - |
| V179E | 91 | 75 | P | EFV, ETR, NVP, RPV | 16 | P | EFV, ETR, NVP, RPV |
| V179VD | 2 | 1 | P | EFV, ETR, NVP, RPV | 1 | P | EFV, ETR, NVP, RPV |
| V179D, K103R | 4 | 4 | I | EFV, ETR, NVP, RPV | 0 | - | - |
| Y181C | 2 | 2 | H | DOR, EFV, ETR, NVP, RPV | 0 | - | - |
| **PIs** | 16 | 13 |  |  | 3 |  |  |
| K43T | 3 | 2 | P | NFV, TPV | 1 | P | NFV, TPV |
| K43KT | 1 | 1 | P | NFV, TPV | 0 | - | - |
| L33F | 1 | 0 | - | - | 1 | P | FPV, NFV, TPV |
| L90M | 1 | 1 | H | ATV, FPV, IDV, LPV, NFV, SQV | 0 | - | - |
| M46L | 1 | 1 | L | NFV | 0 | - | - |
| M46MI | 2 | 1 | I | ATV, FPV, IDV, LPV, NFV, SQV | 1 | I | ATV, FPV, IDV, LPV, NFV, SQV |
| M46ML | 1 | 1 | L | ATV, FPV, IDV, LPV, NFV, SQV, TPV | 0 | - | - |
| N83ND | 1 | 1 | L | ATV, IDV, NFV, SQV, TPV | 0 | - | - |
| Q58E | 5 | 5 | L | NFV, TPV | 0 | - | - |
| **INIs** | 8 | 4 |  |  | 4 |  |  |
| E157Q | 4 | 1 | P | EVG, RAL | 3 | P | EVG, RAL |
| G163R | 1 | 1 | L | RAL | 0 | - | - |
| L74M | 1 | 1 | P | CAB | 0 | - | - |
| L74LM, G149A | 1 | 1 | P | CAB | 0 | - | - |
| S153A | 1 | 0 | - | - | 1 | S | - |

Note: H: High-level resistance; I: Intermediate-level resistance; L: Low-level resistance; P: Potential resistance. ATV: Atazanavir; DRV: Darunavir; FPV: Fosamprenavir; IDV: Indinavir; LPV/r: Lopinavir/ritonavir; NFV: Nelfinavir; SQV: Saquinavir; TPV: Tipranavir; 3TC: Lamivudine; ABC: Abacavir; AZT: Zidovudine; D4T: Stavudine; DDI: Didanosine; FTC: Emtricitabine; TDF: Tenofovir disoproxil fumarate; DOR: Doravirine; EFV: Efavirenz; ETR: Etravirine; NVP: Nevirapine; RPV: Rilpivirine; BIC: Bictegravir; CAB: Cabotegravir; DTG: Dolutegravir; EVG: Elvitegravir; RAL: Raltegravir.
