# Supplementary material for: Analysis of HIV Drug Resistance Mutations Along With Treatment Outcomes of Efavirenz‐Based Regimens in Patients With V179D/E Mutations in Guangzhou, China
Source: Can J Infect Dis Med Microbiol. 2026 Aug 18;2026:2598894. doi: 10.1155/cjid/2598894 (PMC13485334; doi:10.1155/cjid/2598894)
Supplement: Supplementary file 3 [file CJID-2026-2598894-s005.docx]

**Table S3. Comparison of baseline characteristics of individuals with V179D/E mutation across different treatment regimens**

| **Characteristics** | **Non-EFVgroup (n=46)** | **EFV group (n=66)** | **P** |
| --- | --- | --- | --- |
| **Age, median (IQR)** | 32 (25, 38.25) | 30 (24.25, 40.5) | 0.657 |
| **Gender, n (%)** |  |  | 0.882 |
| Male | 45 (97.83%) | 63 (95.45%) |  |
| Female | 1 (2.17%) | 3 (4.55%) |  |
| **Transmission, n (%)** |  |  | 0.617 |
| MSM | 38 (82.61%) | 52 (78.79%) |  |
| HES | 8 (17.39%) | 14 (21.21%) |  |
| **Treatment Duration (year）** | 2.49 (1.57, 2.98) | 1.86 (1.27, 2.13) | 0.050 |
| **HIVRNA (copies/ml)** | 1.36e+05 (6.238e+04, 1.36e+05) | 8.595e+04 (1.478e+04, 1.36e+05) | 0.187 |
| **CD4 level** | 285.50 (191.00, 405.25) | 293.50 (208.00, 424.25) | 0.465 |
| **CD8 level** | 1104.50 (882.25, 1572.50) | 1123.00 (797.00, 1422.00) | 0.073 |
| **Th/Ts** | 0.20 (0.10, 0.39) | 0.26 (0.20, 0.40) | 0.284 |
