# Supplementary material for: Analysis of HIV Drug Resistance Mutations Along With Treatment Outcomes of Efavirenz‐Based Regimens in Patients With V179D/E Mutations in Guangzhou, China
Source: Can J Infect Dis Med Microbiol. 2026 Aug 18;2026:2598894. doi: 10.1155/cjid/2598894 (PMC13485334; doi:10.1155/cjid/2598894)
Supplement: Supplementary file 4 [file CJID-2026-2598894-s006.docx]

**Table S4. Characteristics of patients with V179D/E mutation and drug resistances**

| Cases | Time to drug resistance | HIVRNA*  (Copies/ml) | Mutation sites |
| --- | --- | --- | --- |
| No 1 | 6 Months | 366 | Failure in resistance tests |
| No 2 | 4 Months | 92600 | NNRTI：E138K、V179E、Y81C、H221Y  NRTI：S68G、M184I |
| No 3 | 2 years | 901 | NNRTI： V179E、 Y181C、Y188L  NRTI：K65N |
| No 4 | 6 Months | 10100 | NNRTI：K103N、V179E、H221Y  NRTI：L74LI、M184V |
| No 5 | 6 Months | 61700 | NNRTI：K103N、V179E、M230L  NRTI：K65KR，M184I |
| No 6 | 6 Months | 203000 | NNRTI：V106M、V179D  NRTI：K65R |
| No 7 | 6 Months | 353 | NNRTI：V106M、V179E  NRTI：K65R |

* HIVRNA level at the time point of drug resistance.
